# Supplementary material for: A C57BL/6 Mouse Model of SARS-CoV-2 Infection Recapitulates Age- and Sex-Based Differences in Human COVID-19 Disease and Recovery
Source: Vaccines (Basel). 2022 Dec 25;11(1):47. doi: 10.3390/vaccines11010047 (PMC9860616; doi:10.3390/vaccines11010047)
Supplement: Supplementary file 1 [file vaccines-11-00047-s001.zip › vaccines-2104763-supplementary.pdf]

Supplementary Materials

Figure S1

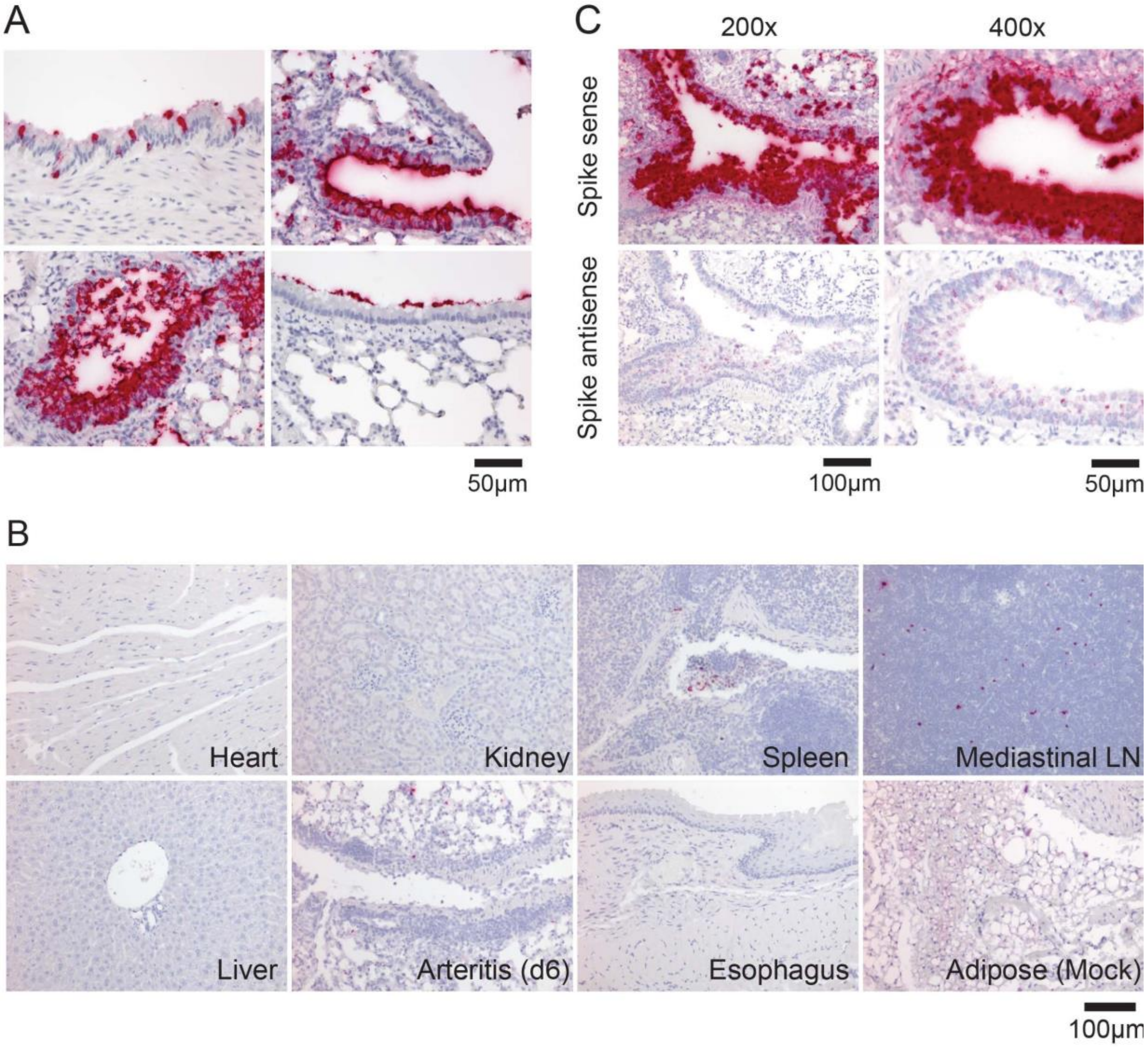

Figure S2

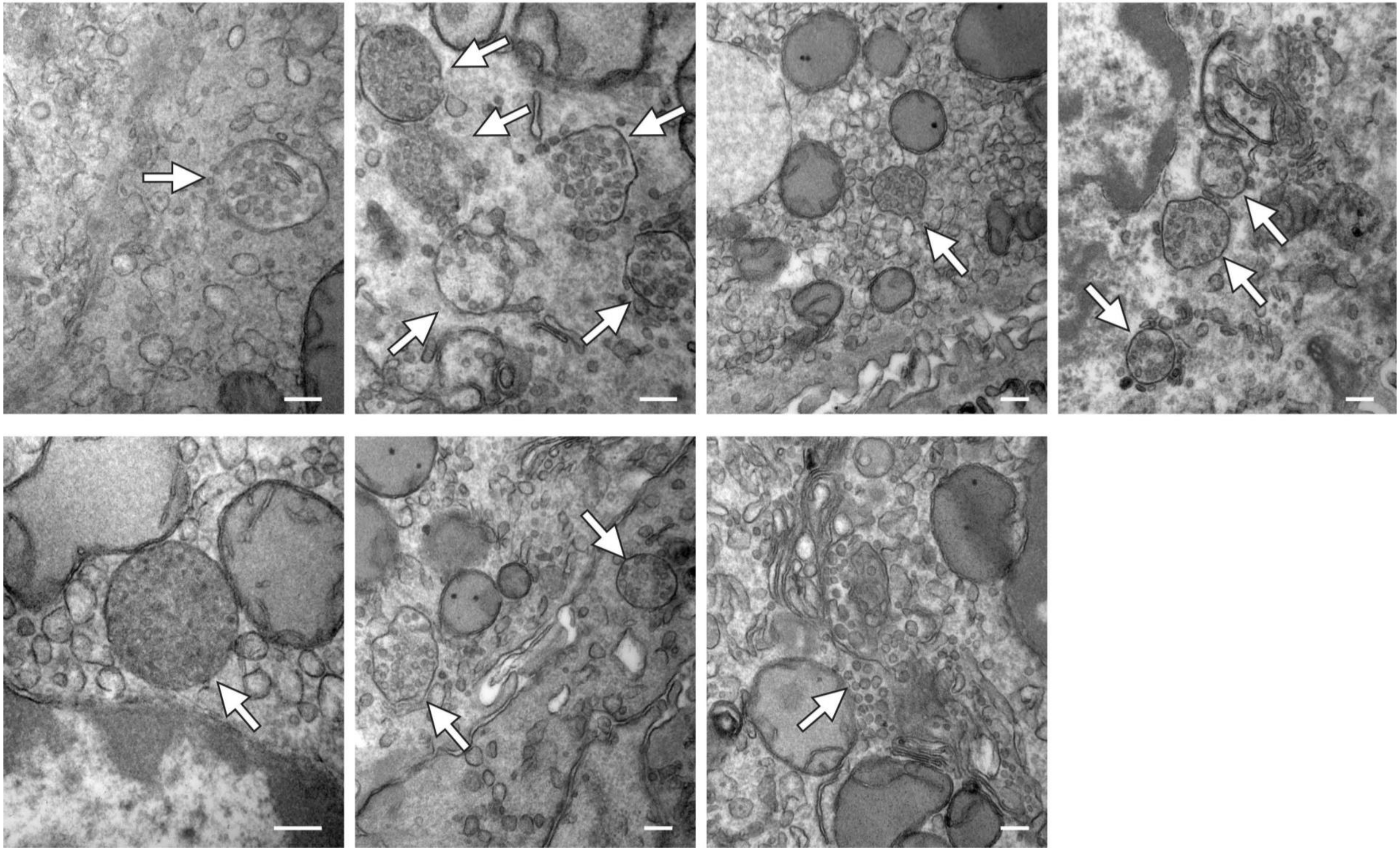

Figure S3

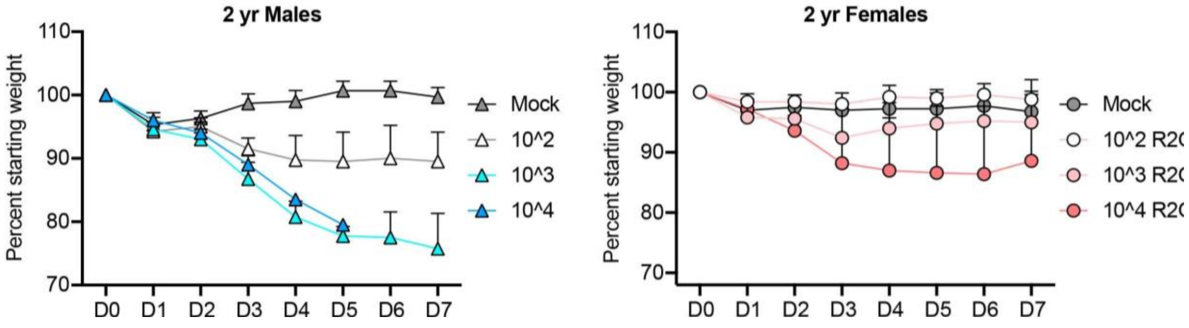
2 yr Males
Percent starting weight
110
100
90
80
70
D0
D1
D2
D3
D4
D5
D6
D7
Mock
10^2
10^3
10^4
2 yr Females
Percent starting weight
110
100
90
80
70
D0
D1
D2
D3
D4
D5
D6
D7
Mock
10^2 R2(
10^3 R2(
10^4 R2(

Figure S4

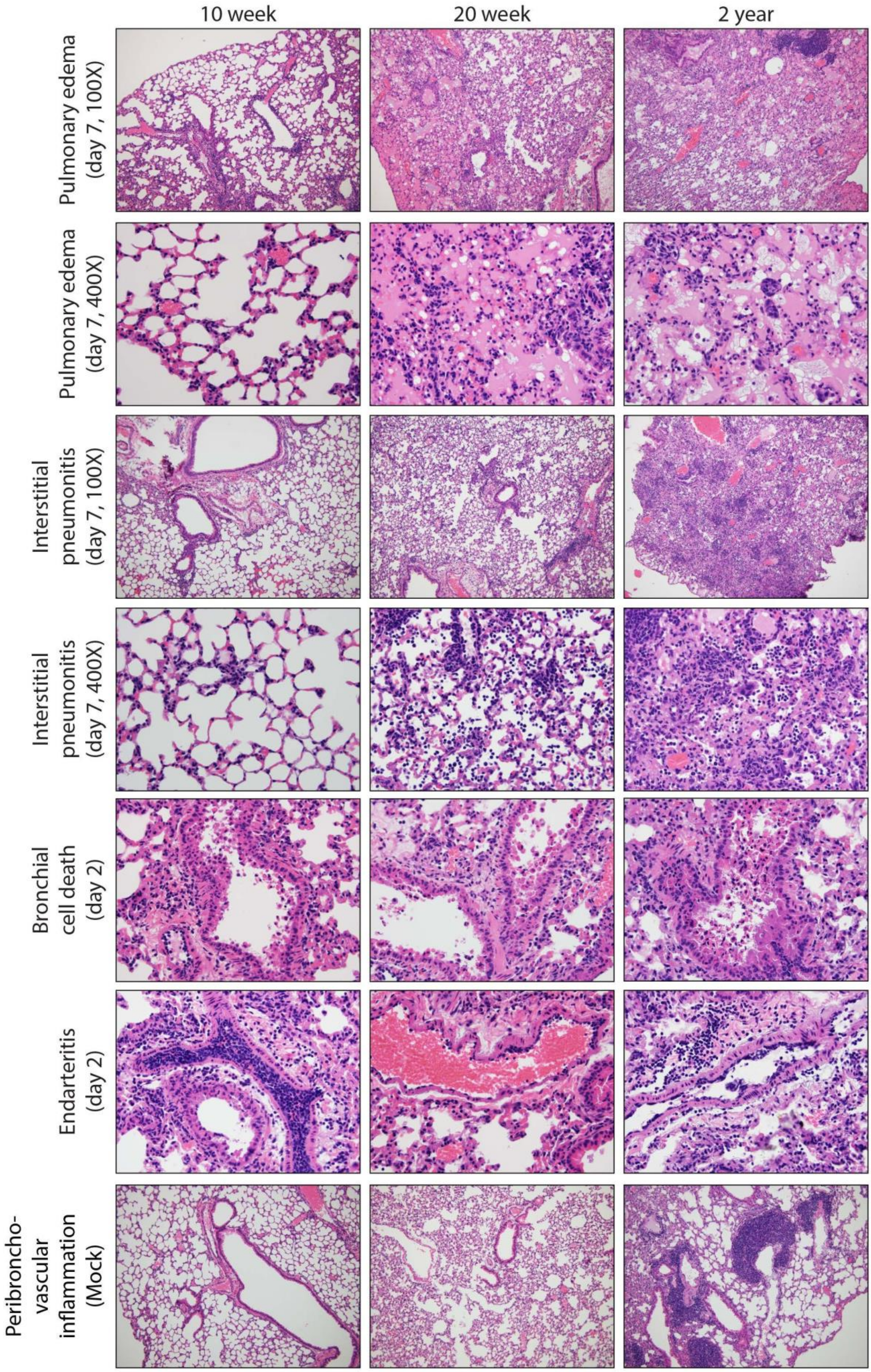
10 week
20 week
2 year
Pulmonary edema (day 7, 100X)
Pulmonary edema (day 7, 400X)
Interstitial pneumonitis (day 7, 100X)
Interstitial pneumonitis (day 7, 400X)
Bronchial cell death (day 2)
Endarteritis (day 2)
Peribroncho-vascular inflammation (Mock)

Figure S5

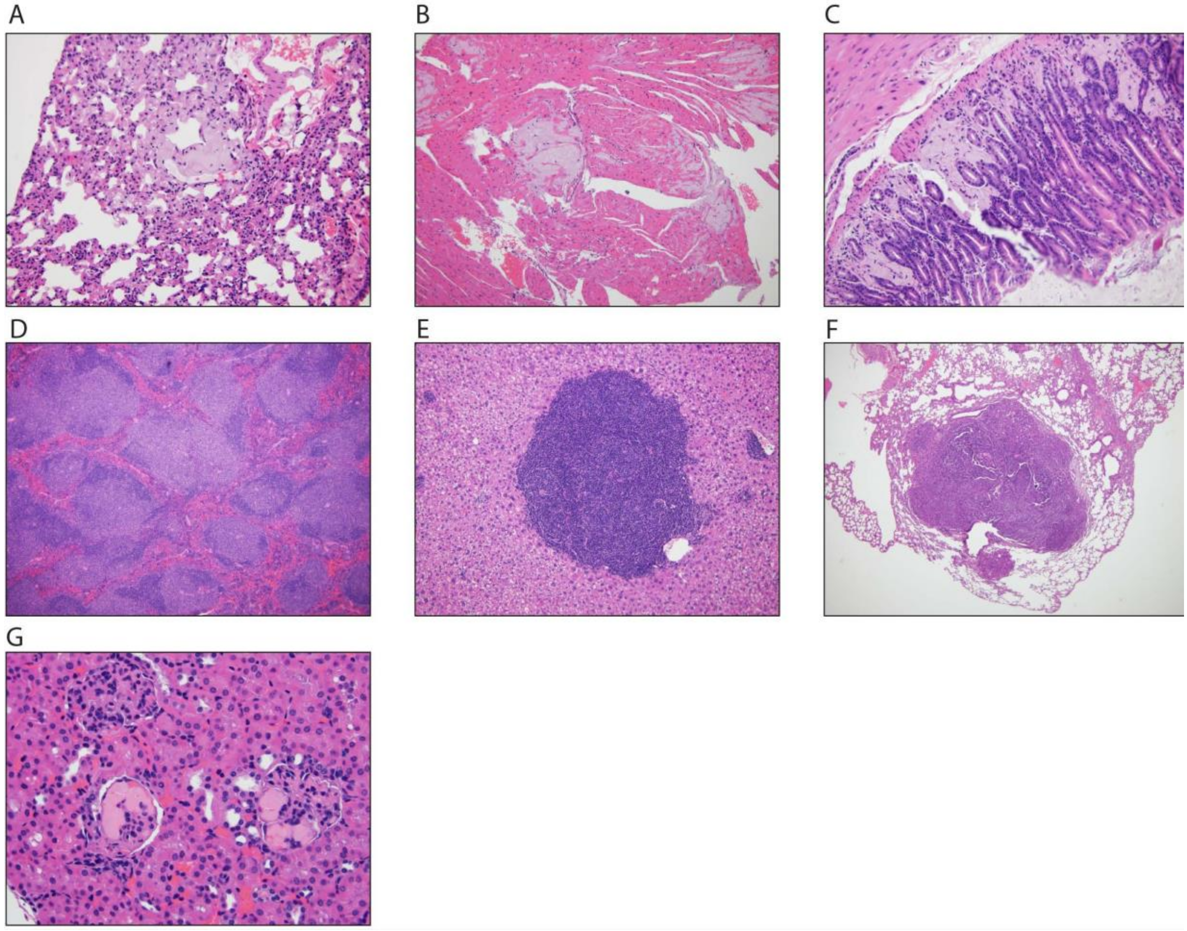

Figure S6

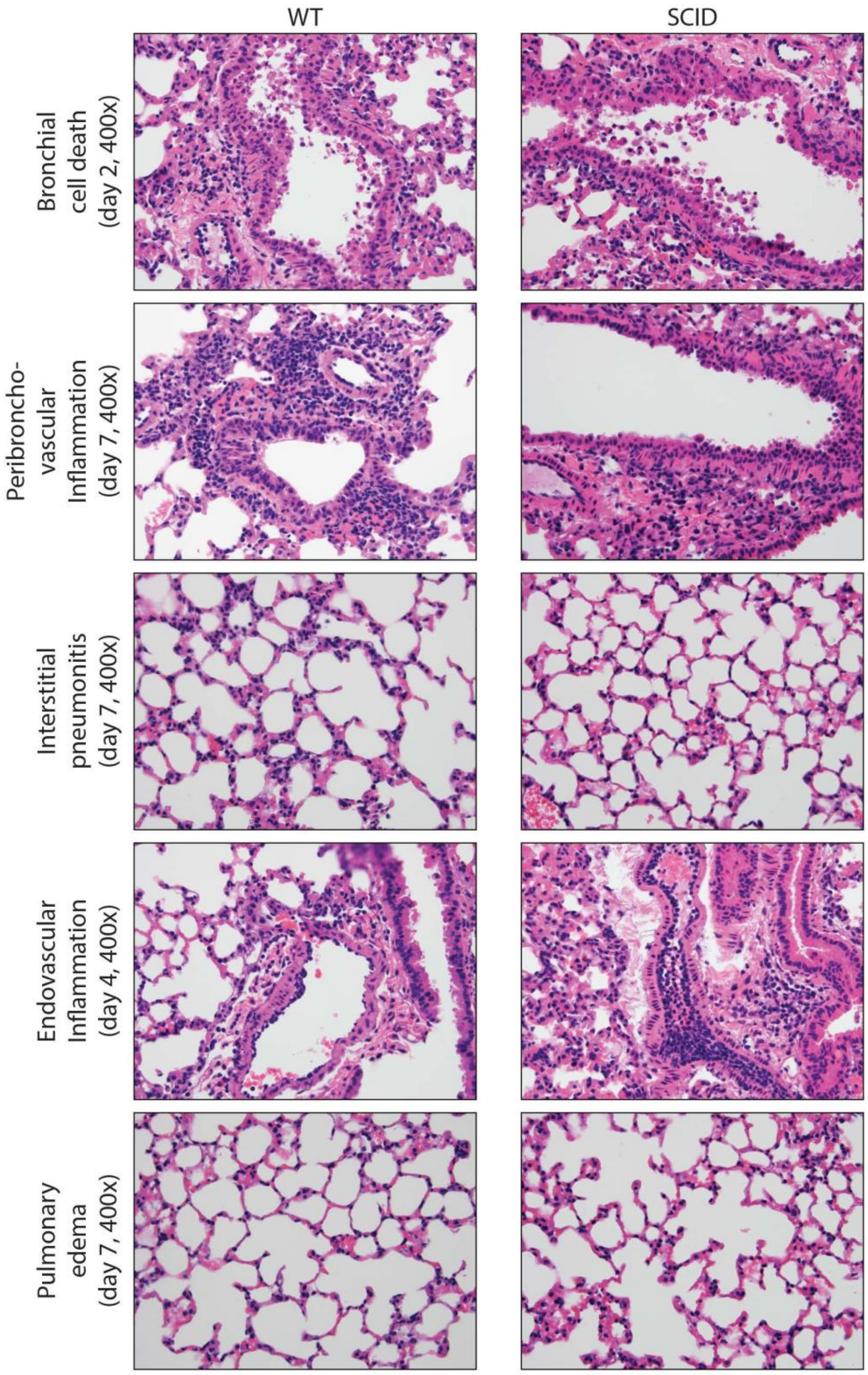
WT
SCID
Bronchial cell death (day 2, 400x)
Peribroncho-vascular Inflammation (day 7, 400x)
Interstitial pneumonitis (day 7, 400x)
Endovascular Inflammation (day 4, 400x)
Pulmonary edema (day 7, 400x)

Figure S7

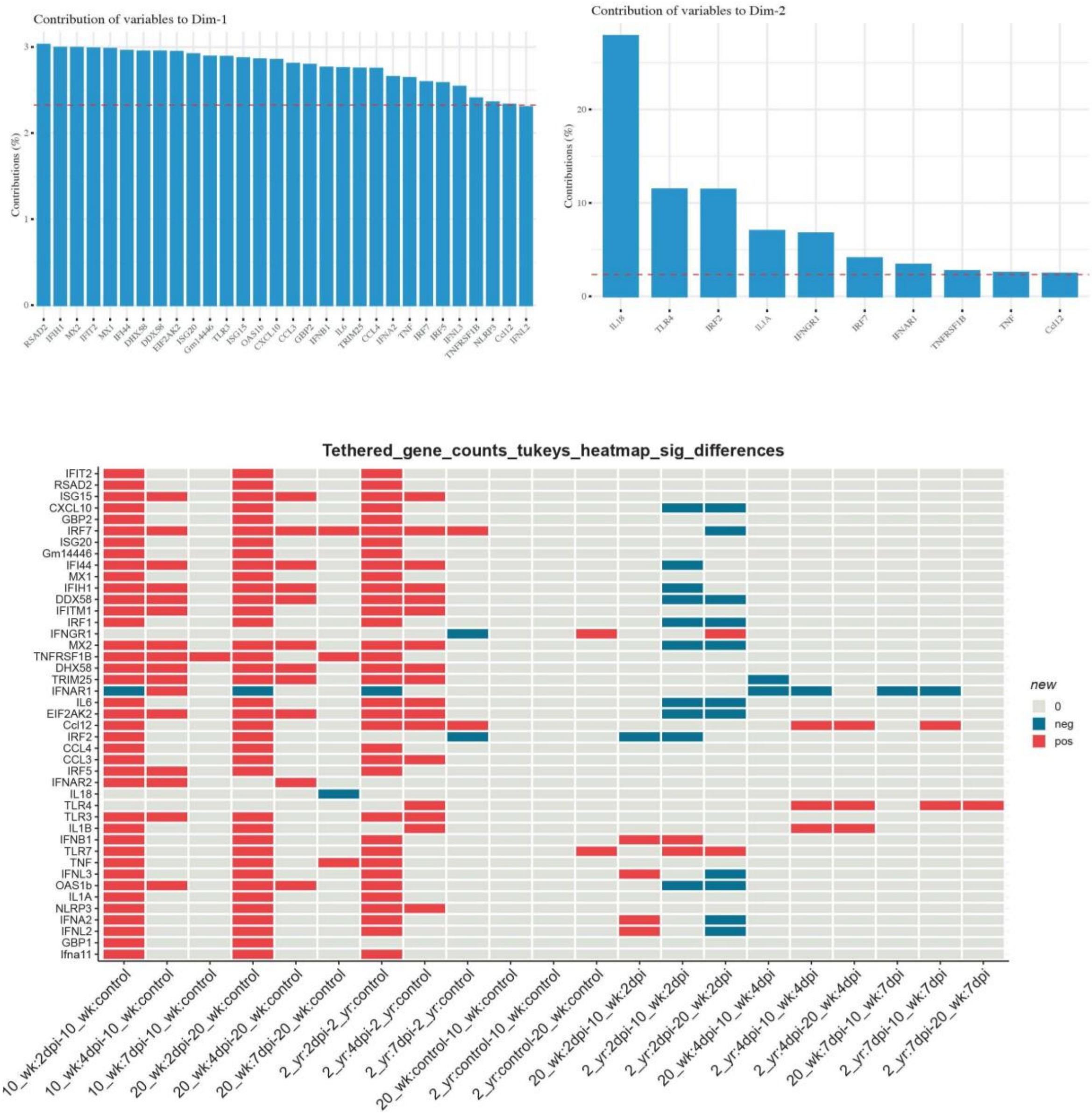

Contribution of variables to Dim-1
Contributions (%)
0
1
2
3
RSAD2
IFIH1
MX2
IFIT2
MX1
IFI44
DHX58
DDX58
EIF2AK2
ISG20
Gm14446
TLR3
ISG15
OAS1b
CXCL10
CCL3
GBP2
IFNB1
IL6
TRIM25
CCL4
IFNA2
TNF
IRF7
IRF5
IFNL3
TNFRSF1B
NLRP3
Ccl12
IFNL2
Contribution of variables to Dim-2
Contributions (%)
0
10
20
IL18
TLR4
IRF2
IL1A
IFNGR1
IRF7
IFNAR1
TNFRSF1B
TNF
Ccl12
Tethered_gene_counts_tukeys_heatmap_sig_differences
IFIT2
RSAD2
ISG15
CXCL10
GBP2
IRF7
ISG20
Gm14446
IFI44
MX1
IFIH1
DDX58
IFITM1
IRF1
IFNGR1
MX2
TNFRSF1B
DHX58
TRIM25
IFNAR1
IL6
EIF2AK2
Ccl12
IRF2
CCL4
CCL3
IRF5
IFNAR2
IL18
TLR4
TLR3
IL1B
IFNB1
TLR7
TNF
IFNL3
OAS1b
IL1A
NLRP3
IFNA2
IFNL2
GBP1
Ifna11
10_wk:2dpi-10_wk:control
10_wk:4dpi-10_wk:control
10_wk:7dpi-10_wk:control
20_wk:2dpi-20_wk:control
20_wk:4dpi-20_wk:control
20_wk:7dpi-20_wk:control
2_yr:2dpi-2_yr:control
2_yr:4dpi-2_yr:control
2_yr:7dpi-2_yr:control
20_wk:control-10_wk:control
2_yr:control-10_wk:control
2_yr:control-20_wk:control
20_wk:2dpi-10_wk:2dpi
2_yr:2dpi-10_wk:2dpi
2_yr:2dpi-20_wk:2dpi
20_wk:4dpi-10_wk:4dpi
2_yr:4dpi-10_wk:4dpi
2_yr:4dpi-20_wk:4dpi
20_wk:7dpi-10_wk:7dpi
2_yr:7dpi-10_wk:7dpi
2_yr:7dpi-20_wk:7dpi
new
0
neg
pos

Figure S8

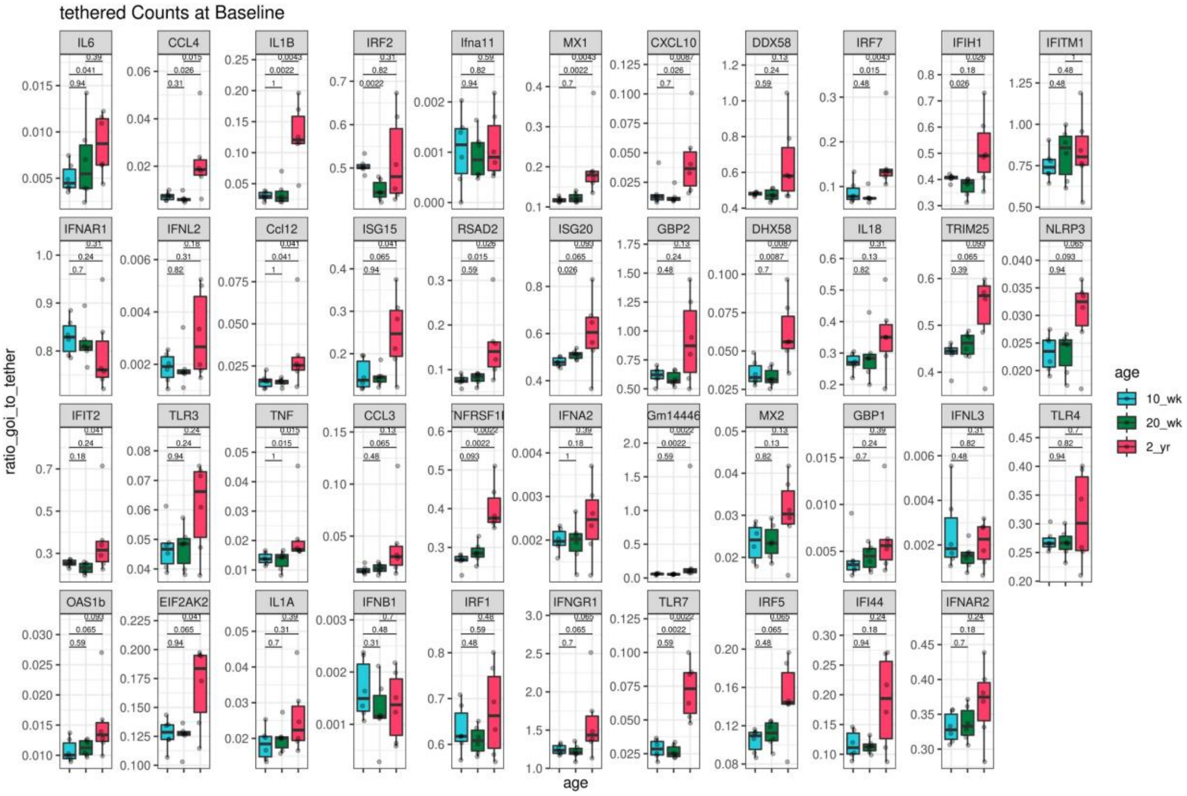

Figure S9

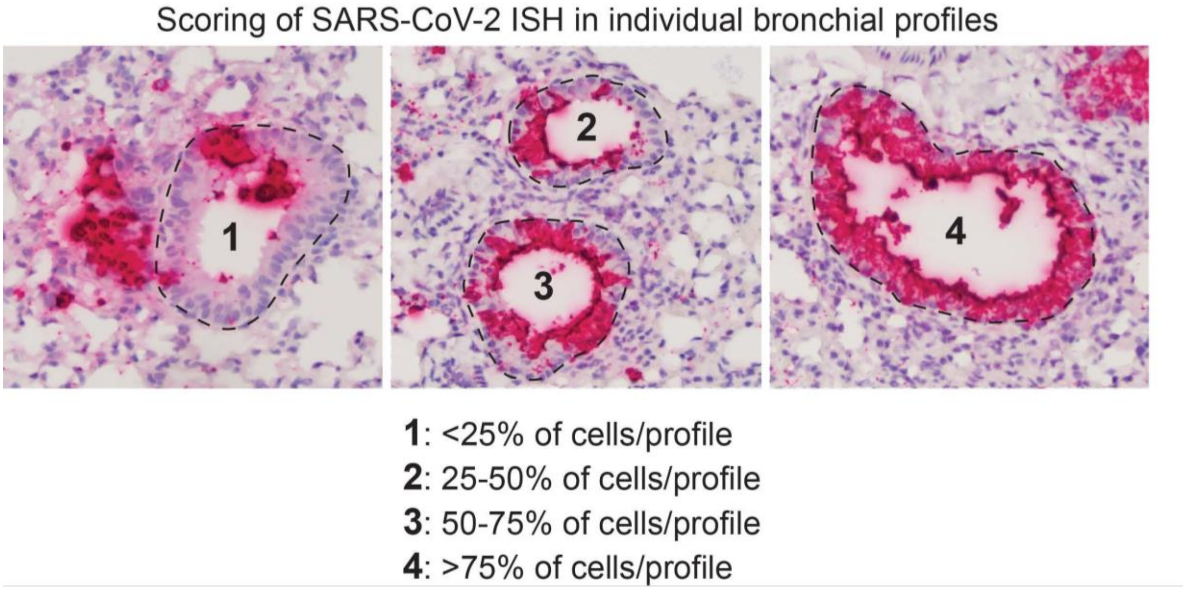

Figure S10

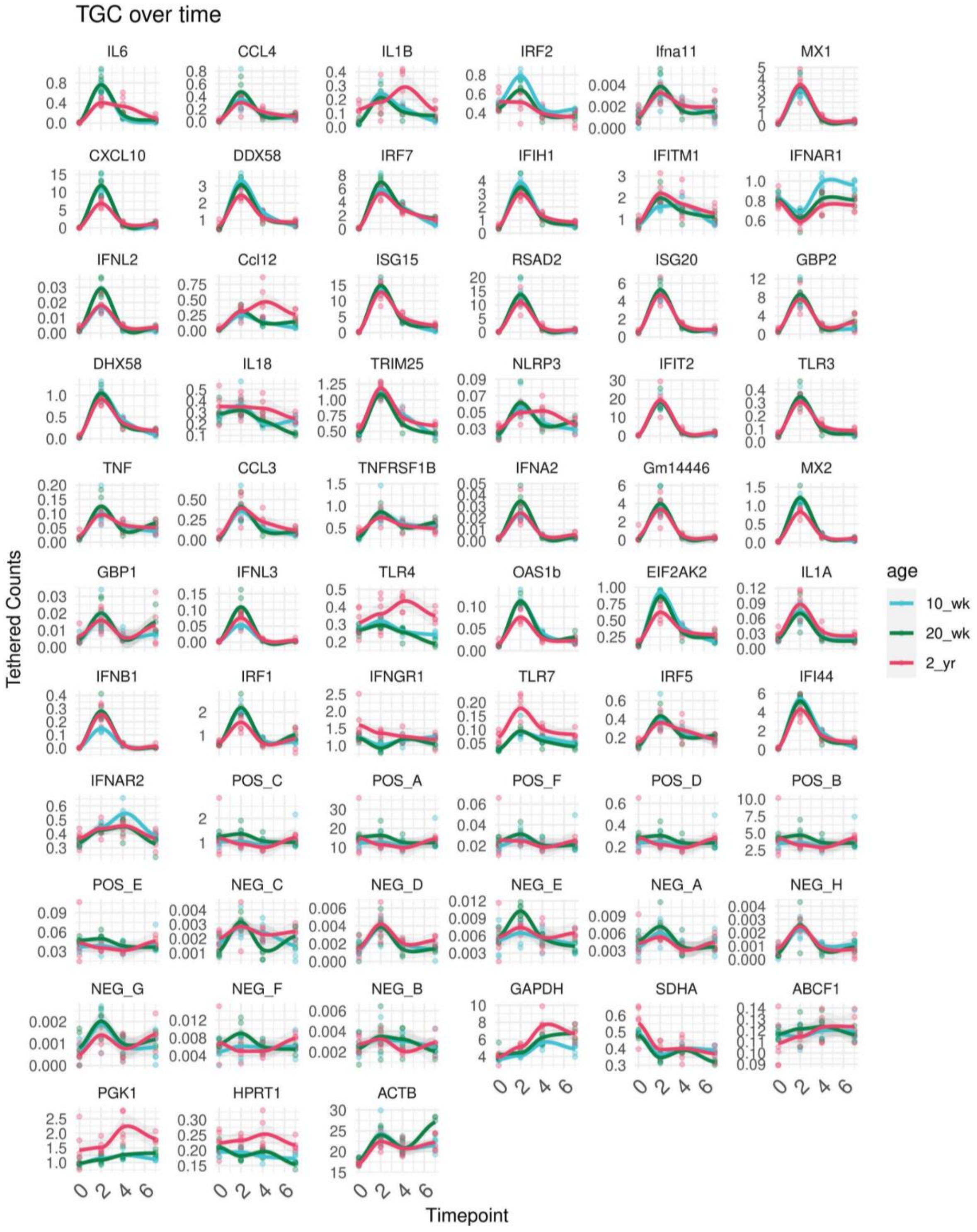
TGC over time
IL6
CCL4
IL1B
IRF2
Ifna11
MX1
CXCL10
DDX58
IRF7
IFIH1
IFITM1
IFNAR1
IFNL2
Ccl12
ISG15
RSAD2
ISG20
GBP2
DHX58
IL18
TRIM25
NLRP3
IFIT2
TLR3
TNF
CCL3
TNFRSF1B
IFNA2
Gm14446
MX2
GBP1
IFNL3
TLR4
OAS1b
EIF2AK2
IL1A
IFNB1
IRF1
IFNGR1
TLR7
IRF5
IFI44
IFNAR2
POS_C
POS_A
POS_F
POS_D
POS_B
POS_E
NEG_C
NEG_D
NEG_E
NEG_A
NEG_H
NEG_G
NEG_F
NEG_B
GAPDH
SDHA
ABCF1
PGK1
HPRT1
ACTB
Tethered Counts
Timepoint
age
10_wk
20_wk
2_yr
